# Oleamide-Mediated Polarization of M1 Macrophages and IL-1β Production by Regulating NLRP3-Inflammasome Activation in Primary Human Monocyte-Derived Macrophages

**DOI:** 10.3389/fimmu.2022.856296

**Published:** 2022-04-19

**Authors:** Prapakorn Wisitpongpun, Pachuen Potup, Kanchana Usuwanthim

**Affiliations:** Cellular and Molecular Immunology Research Unit (CMIRU), Faculty of Allied Health Sciences, Naresuan University, Phitsanulok, Thailand

**Keywords:** MDMs, monocyte-derived macrophages, macrophage - cell, polarization, oleamide (OA), inflammasome

## Abstract

Macrophages are a type of innate immune cell that activates the NLRP3 inflammasome, causing the release of the cytokine IL-1β, which is a crucial mediator of the inflammatory response. NLRP3 activation that is dysregulated worsens a variety of inflammatory and autoimmune diseases, as well as neurodegenerative diseases. Oleamide is an endogenous fatty acid amide that was first determined as a sleep-inducing molecule and later shown to have wide-ranging beneficial effects on the central nervous system. How oleamide influences human macrophage polarization and NLRP3-inflammasome activation remains unclear. The effect of oleamide on macrophage polarization was explored using an *in vitro* culture of primary human monocyte-derived macrophages (MDMs) supplemented with human serum-containing media. Cellular and molecular mechanisms of oleamide-regulated MDMs polarization were also investigated. Results showed that oleamide promoted naïve macrophages (M0) toward the M1 phenotype by upregulating M1-associated genes (*IL-1β*, *iNOS*, *CXCL10*), along with downregulation of M2-associated genes (*Arg-1*, *CD206*, *CCL22*). Cell surface expression indicated that oleamide enhanced CD80 expression in M0 naïve macrophages and hider CD206 and CD163 expression in M2 macrophages. Higher production of IL-1β cytokine was observed but with no alteration in IL-6 and TNF-α levels by MDMs and differentiated THP-1 models. Whether oleamide functioned as a second signal that activated the NLRP3 inflammasome and mediated IL-1β production was further investigated using LPS-primed MDMs followed by oleamide treatment that induced activation of inflammasome-related proteins including NLRP3, ASC, cleaved casp-1, and cleaved IL-1β. These findings suggested that oleamide promoted M1 macrophage polarization and increased IL-1β production by activating the NLRP3 inflammasome in primary MDMs. This research reveals a new function for oleamide as well as prospective targets for treating NLRP3-related inflammatory disorders.

**Graphical Abstract:**
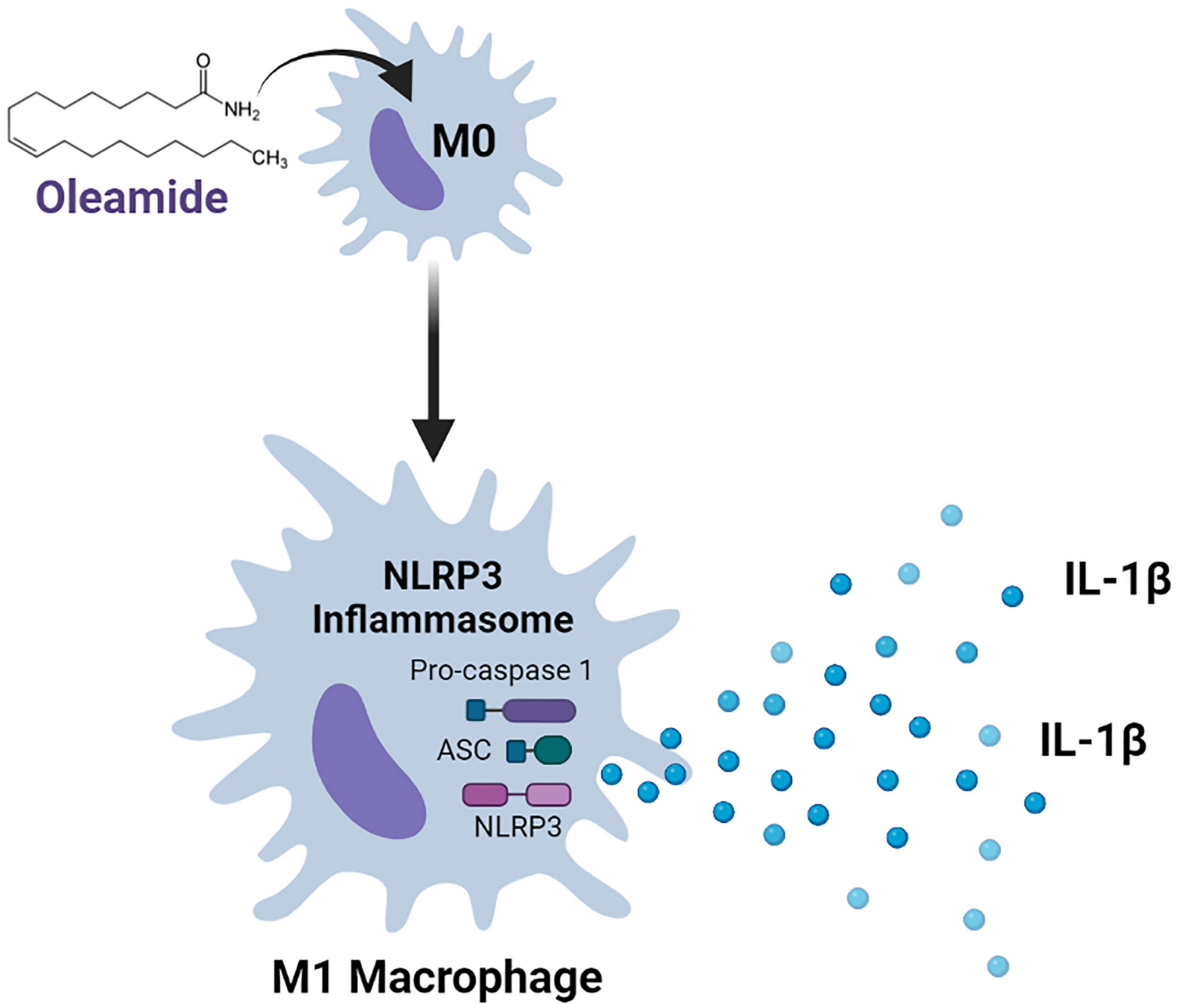


## Introduction

Macrophages comprise a heterogeneous population of the innate immune system involved in several processes of health and disease (1–3). The heterogeneity of macrophages is commonly referred to as polarization, a process by which macrophages display different functional phenotypes in response to specific microenvironmental stimuli and signals (4). Macrophage polarization is conventionally divided into three groups as naïve macrophages (MØ; also called M0), which readily differentiate into two major phenotypes as classically activated macrophages (M1) and alternatively activated macrophages (M2) (1–3). M1 macrophages, also known as pro-inflammatory macrophages, are characterized by high levels of reactive oxygen species (ROS) and nitric oxide (NO) and the production of pro-inflammatory cytokines (1, 5). Inflammation and microbicidal activity are linked to M1 macrophages (1, 5). M2 macrophages, commonly known as anti-inflammatory macrophages, have multiple receptors expressed, including the mannose receptor (MRC1), scavenging receptor CD163, dectin-1, and DC-SIGN (1, 5). Immunosuppression, wound repair, and tumor promotion are all mechanisms related with M2 macrophages (3).

Interleukin-1β (IL-1β) is a pro-inflammatory cytokine produced mainly by innate immune cells as a response to infection and injury (6). In macrophages, NLRP3 inflammasome-mediate IL-1β production can be primed by TLRs activation (Signal 1), which activates NF-κB or a non-NF-κB pathway to produce pro-IL-1β and pro-IL-18. The second signal is the oligomerization of NLRP3, pro-caspase 1, and ASC (apoptosis-associated speck-like protein containing a caspase recruitment domain), an adapter protein (7). The assembly of these proteins activates caspase-1, which then converts pro-IL-1β and pro-IL-18 into mature IL-1β and IL-18 that are released, resulting in inflammatory reactions (8, 9). As a result, dysregulation of the NLRP3 inflammasome has been linked to the advancement of a number of inflammatory and autoimmune disorders, including neurodegenerative diseases like Alzheimer’s, Parkinson’s, and amyotrophic lateral sclerosis (10–12). This shows that targeting the NLRP3 inflammasome as a therapeutic strategy for treating NLRP3-related inflammatory disorders may be feasible.

Oleamide (Cis-9,10-octadecenamide) is an endogenous fatty acid discovered in the cerebrospinal fluid of sleep-deprived cats and later identified as an endogenous sleep-inducing agent (13). Oleamide has a variety of effects on the central nervous system, including antiepileptic (14), memory regulation (15, 16), and hypothermia elicitation (17). In addition, oleamide has been shown to decrease LPS-induced iNOS and COX-2 production in BV2 murine microglial cells through inhibiting NF-κB activation (18). Oleamide has recently been identified as a dual-active component that both decreases amyloid-β (Aβ) accumulation through increased microglial phagocytosis and suppresses LPS-induced microglial inflammation by reducing TNF-α and MIP-1α production (19). These findings in microglial cells suggested that oleamide might affect macrophage activity, although it is still uncertain how oleamide influences macrophage polarization and the NLRP3 inflammasome.

This study investigated the regulatory effects of oleamide on the progression of M1/M2 macrophage polarization using an *in vitro* culture of primary human monocyte-derived macrophages (MDMs) as a model. Results showed that oleamide promoted naïve macrophages (M0) toward the M1 phenotype and hider development of the M2 phenotype. Moreover, oleamide critically regulates IL-1β production in MDMs by stimulating the NLRP3 inflammasome.

## Material and Methods

### Isolation of Human MDMs

MDMs were isolated from a blood buffy coat of healthy blood donors. Briefly, buffy coats were transferred into 50 ml tubes and centrifuged at 3,000 rpm for 30 min. The WBC layer was collected and then diluted 1:1 in PBS-EDTA (1 mM). Diluted WBCs were gently overlaid on top of Ficoll-Paque solution (density 1.077 g/ml, GE Healthcare, Chicago, USA) at a 1:1 ratio and then centrifuged at 3,000 rpm for 30 min. The peripheral blood mononuclear cell (PBMC) layer at the interface was transferred to a new 50 ml tube and washed once with 40 ml PBS-EDTA by centrifugation at 1500 rpm for 5 min. The PBMCs were then diluted in 15 ml PBS-EDTA and overlaid on 46% Percoll solution (density 1.131 g/ml, GE Healthcare, Chicago, USA) at a 1:1 ratio followed by centrifugation at 3,000 rpm for 30 min. Then monocyte layer at the interface was collected into a new 50 ml tube, washed 3-5 times in 40 ml PBS-EDTA, and cultured in desired conditions. The purity of MDMs on days 0-6 was shown in **Supplementary Figure 1**.

### MDMs Culture and Polarization

MDMs were cultured in RPMI 1640 supplemented with 10% human serum from buffy coats of healthy donors and 1% antibiotic-antimycotic (Gibco™, USA #15240062) at 37°C with 5% CO_2_. The M0 macrophages condition, monocytes were grown in a complete medium without stimulation for 6 days. In the M1 macrophages, monocytes were cultured in the presence of 50 ng/ml of GM-CSF (ImmunoTools GmbH, Germany) for 6 days. In the M2 macrophages, monocytes were cultured in 50 ng/ml M-CSF (ImmunoTools GmbH, Germany) for 6 days. Cell culture mediums were replaced every 3 days in all conditions. On day 6, M0 macrophages were incubated in complete medium alone for 24 h to serve as naïve macrophage or negative control cells. M1 macrophages were polarized with 20 ng/ml IFN-γ (ImmunoTools GmbH, Germany) and 10 ng/ml LPS of *Escherichia coli* O55:B5 (Sigma Aldrich, MO, USA) for 24 h. M2 macrophages were polarized using 20 ng/ml IL-4 (ImmunoTools GmbH, Germany) for 24 h. For experimental groups, M0, M1, and M2 macrophages (day 6) were cultured in desired conditions plus oleamide for 24 h.

### THP-1 Cell Culture and Differentiation

THP-1 cells obtained from ATCC were maintained in RPMI-1640 supplemented with 10% FBS and 1% Antibiotic-Antimycotic (Gibco™, USA #15240062) at 37°C with 5% CO_2_. THP-1 were differentiated into macrophages by incubating with 100 nM phorbol-12-myristate-13-acetate (PMA) (Sigma-Aldrich, St. Louis, MO, USA) for 24 h and then replaced with PMA-free complete medium for 72 h. Following differentiation, cells were polarized into M1 macrophages by incubation with 20 ng/ml IFN-γ (ImmunoTools GmbH, Germany) and 10 ng/ml LPS (Sigma Aldrich, MO, USA) for 24 h. M2 macrophages were obtained by incubation with 20 ng/ml IL-4 (ImmunoTools GmbH, Germany) for 24 h. M0 macrophages were incubated with a complete medium only for 24. For experimental groups, M0, M1, and M2 macrophages were cultured in desired conditions plus oleamide for 24 h.

### Inflammasome Activation

Isolated monocytes (Day 0) were seeded at a density of 4 x 10^5^ cells/well in 12-well plates and differentiated for 6 days in complete medium. Cell culture medium was replaced every 3 days. On day 6, MDMs were stimulated with 100 ng/ml LPS for 3 h. Cells were then stimulated with oleamide (10-40 µg/ml) (Sigma Aldrich, St. Louis, MO, USA) or adenosine triphosphate (ATP) (Sigma-Aldrich, St. Louis, MO, USA) as the positive control for 1 and 3 h. After stimulation, supernatants were collected, and cytokine levels were measured using an ELISA. Cells were collected, and qRT-PCR was used to examine mRNA expression.

### Cell Viability Assay

Cell viability was determined using the Methyl Thiazol Tetrazolium (MTT) assay. MDMs (Day 6) were seeded at a density of 2.0 x 10^4^ cells/well in a 96-well plate and incubated with the serial concentration of oleamide for 48 h. The MTT salt solution (Thermo Fisher Scientific, Waltham, MA, USA) was then added and incubated for 3 h at 37°C. To dissolve the formazan crystal, 100 µl of dimethyl sulfoxide (DMSO) (VWR International, West Chester, PA, USA) was added to each well. Plates were gently shaken for 5-10 min. The absorbance was measured at 570 nm using a microplate reader (PerkinElmer, Inc., USA).

### Flow Cytometry

After stimulation, MDMs were harvested using Trypsin-EDTA for 15 min followed by a cell scraper. Cells were washed once with ice-cold PBS and resuspended in ice-cold FACS buffer (0.5% BSA and 0.05% sodium azide in PBS). Cells (2 x 10^5^ cells) were stained with the fluorochrome-conjugated anti-human antibodies, including CD80-FITC, CD163-PE, and CD206-FITC (BioLegend, San Diego, CA, USA), CD14-PE, CD16-FITC, and isotype control (ImmunoTools GmbH, Germany) for 30 min at 4°C in FACS buffer. The cells were then washed three times in ice-cold PBS and fixed with 2% formaldehyde (Sigma Aldrich, MO, USA) at 4°C for 10 min. After fixation, cells were washed once in PBS, resuspended in FACS buffer, and stored at 4°C in the dark until used. Cell surface expressions were detected using CytoFlex S (Beckman Coulter Life Sciences, Indiana, USA) and analyzed with CytoExpert software.

### Reverse Transcription-Quantitative Real-Time PCR (RT-qPCR)

Total RNA was extracted using Trizol reagent (Invitrogen, Carlsbad, CA, USA) according to the manufacturer’s instructions. cDNA was synthesized using Tetro cDNA Synthesis Kit (Bioline, Tennessee, USA). The resulting cDNAs were amplified with different primers (**Supplementary Table 1**) using the SensiFAST™ SYBR ^®^ No-ROX Kit (Bioline USA Inc., Taunton, MA, USA). Results were analyzed using the relative gene expression method. Briefly, the relative expression of the target genes was calculated by relating the Ct-value of the target gene to unstimulated M0 macrophage. The quantitative cycle (Ct) values were used to calculate relative variations in gene expression between groups. These values were normalized to a housekeeping gene (β-actin) in the same sample (ΔCt) and expressed as the fold-change over control (2^−ΔΔCt^). Real-time fluorescence detection was performed using a CFX96 Touch Real-Time PCR Detection System (Bio-Rad).

### Scanning Electron Microscope (SEM)

MDM cells were plated into 12-cell well plate containing sterile coverslips. After differentiation, samples were fixed with 4% paraformaldehyde and 2.5% glutaraldehyde in 0.1 M phosphate buffer (1.14 g NaH_2_PO_4_, 1.69 g Na_2_HPO_4_ in a 100-ml final volume of ddH_2_O, pH 7.4) for 30 min at 4°C. Samples were washed three times for 5 min each in 0.1 M phosphate buffer and then dehydrated with graded ethanol (EtOH) series beginning with 25%, 50%, 75%, 95%, and 100% EtOH (5 min for each). Then, the cells were further dehydrated with 1:1 [Hexamethyldisilazane (HMDS): ETOH] for 5 min and 100% HMDS two times for 5 min at room temperature. Samples were then coated with gold particles and imaged with an Apreo 2 SEM (Thermo Fisher Scientific) using a 5 kV incident beam.

### Enzyme-Linked Immunosorbent (ELISA) Assay

Levels of TNF-α, IL-6, IL-1ß and IL-18 cytokines were measured by a sandwich ELISA Kit according to the manufacturer’s instructions (Sino Biological Inc., China). Absorbance of the reaction was measured at 450 nm using an EnSpire^®^ Multimode microplate reader (PerkinElmer, Inc., MA, USA).

### LDH Assay

The release of lactate dehydrogenase (LDH) was measured using the CyQUANT LDH Cytotoxicity Assay (Thermo Fisher, Waltham, USA). MDMs were plated into a 96-well plate at a density of 2.0 x 10^4^ cells/well overnight, and then incubated with LPS for 3 h followed by oleamide (10 - 40 µg/ml) or ATP (30 mM) for 3 h. After incubation, 50 µl of culture medium was transferred to a new 96-well plate and 50 µl of LDH reaction mixture was added, mixed, and incubated for 30 min at room temperature. Then, 50 µl of stop solution was added to terminate the reactions. Absorbances of the reaction mixtures were measured at 490 nm and 680 nm.

### Western Blot Analysis

Cells were washed with ice-cold PBS and lysed with RIPA buffer containing 1X Protease/Phosphatase Inhibitor Cocktail (Sigma Aldrich, St. Louis, MO, USA) for 30 min on ice. The lysate was then transferred into microcentrifuge tubes and centrifuged at 12,000 rpm for 10 min under 4°C. The supernatant containing whole protein was collected and measured for protein concentration by the Bradford assay. Equal amounts of each sample were loaded and separated in 8-12% of SDS-PAGE at 100 V for 1.30 h. Protein was then transferred onto PVDF membrane at 100 V for 1.30 h in an icebox. The membranes were blocked with TBST containing 5% BSA for 1 h and subsequently incubated with the appropriate primary antibodies; Rabbit mAb against IL-1β, cleaved- IL-1β, caspase-1, cleaved caspase-1, NLRP3, ASC/TMS1, P2X7 receptor (Cell Signaling Technology, MA, USA) at 4°C overnight. Membranes were washed 3-5 times in TBST for 5 min each and then incubated with Anti-rabbit IgG, HRP-linked Antibody (Cell Signaling Technology, MA, USA) for 1 h at RT. After incubation, membranes were washed in TBST 3-5 times for 5 min each and the blots were developed by chemiluminescence solution for 1 min. Then, the protein was detected using ChemiDoc™ Touch Imaging System (Bio-Rad, Hercules, CA, USA).

### Statistical Analysis

All data were expressed as mean ± standard deviation (n = 3). For comparisons of more than two groups, one-way ANOVA was performed with multiple comparison correction (Dunnett test) using GraphPad Prism 6.0 software (GraphPad Software Inc., San Diego, CA, USA). p-values < 0.05 were considered statistically significant.

## Results

### Generation of *In Vitro* Culture and Polarization of MDMs

**Figure 1:**
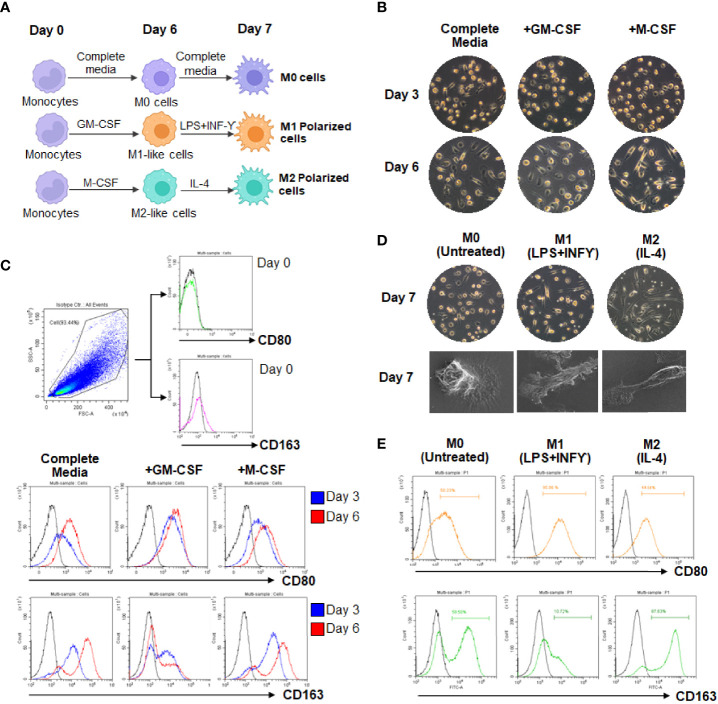
Morphologic and phenotypic characterization of cultured MDMs. **(A)** Scheme of monocyte-derived macrophages (MDMs) culture and polarization. **(B, C)** Differentiation of MDMs at day 3 and 6. **(D)** Polarization of MDMs in complete medium containing M1 (LPS plus IFN-γ)- or M2 (IL-4). (E) Flow cytometry analysis of CD80 and CD163 expressions in culture MDMs at day 7.

To imitate naturally occurring macrophages, monocytes were freshly isolated from the blood buffy coat of healthy donors. MDMs were cultured in complete medium containing human serum alone (M0 cells), or complete medium containing GM-CSF (M1-like cells) or M-CSF (M2-like cells) for 6 days. MDMs were further cultivated for 24 h in the same medium (M0 macrophage) or in the presence of LPS plus IFN-γ (M1 macrophage) or IL-4 (M2 macrophage) (**Figure 1A**). The presence of GM-CSF led to a majority of round adhered with short-elongated shape, whereas the presence of M-CSF enhanced more elongation. MDMs cultured in complete medium containing human serum alone exhibited dual morphology (round adherend and elongated shape), indicating the mixed phenotypes in the M0 macrophages (**Figure 1B** and **Supplementary Figure 3**). Differentiation of MDMs at days 0-6 were analyzed by flow cytometry using CD80 (M1 marker) and CD163 (M2 marker). Results showed that M1-like cells or the presence of GM-CSF upregulated CD80 expression, correlating with downregulation of CD163 at days 0-6, respectively. By contrast, M2-like cells displayed upregulation of CD163 expression with downregulation of CD80. MDMs cultured in a medium containing human serum alone displayed increased expression of CD163 rather than CD80 on the cell surface **(** **Figure 1C** **)**. For fully polarized macrophages, LPS plus IFN-γ treated cells displayed strong induction of CD80 and downregulation of CD163 compared to unpolarized M0 cells. Conversely, IL-4-treated cells displayed high expression of CD163 and low levels of CD80 expression (**Figure 1E**). By using an inverted microscope and a SEM, the exhibit morphology of fully M0, M1, and M2 (**Figure 1D**). Collectively, these data confirmed the M1 and M2 polarization state of LPS plus IFN-γ and IL-4-treated MDMs.

### Oleamide Mediated M1 Macrophages Polarization and IL-1β Production

**Figure 2:**
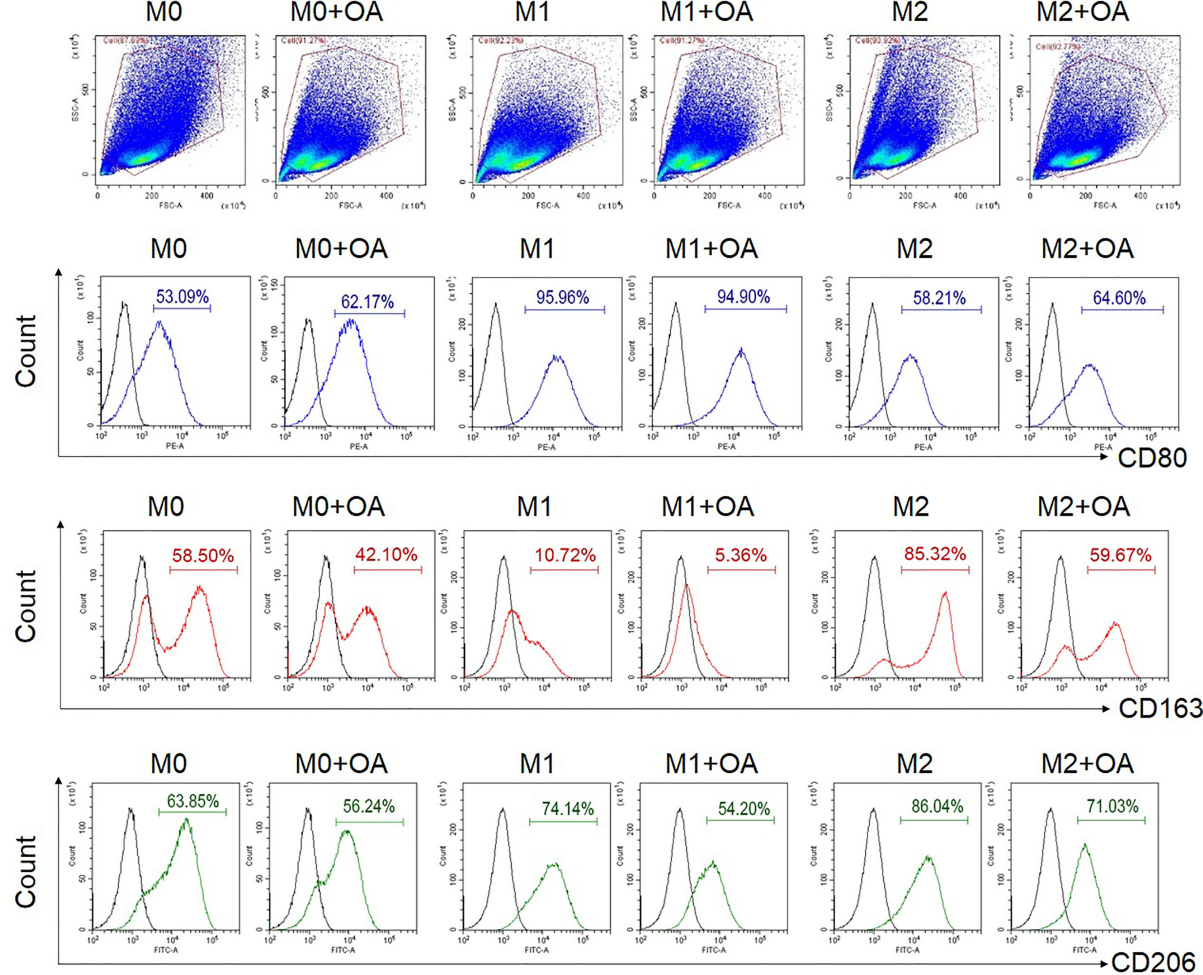
Flow cytometry analysis of surface expressions. MDMs at day 6 were cultured in complete medium only (M0), LPS + INF-γ (M1), or IL-4 (M2) in the presence or absence of oleamide (15 µg/ml) for 24h. LPS, lipopolysaccharides; OA, oleamide.

To investigate the effect of oleamide on macrophage polarization, MDMs were cultured in conditioned media for M1 (LPS + IFN-γ), M2 (IL-4), and M0 (untreated) in the presence or absence of oleamide. These cells were characterized in different conditions based on surface markers, gene expression, and cytokine production. The presence of oleamide enhanced greater elongation as spindle-like shapes of the M0 and M1 macrophages (**Supplementary Figure 4**). Surface expressions were analyzed by flow cytometry using polarization markers including CD80 for the M1 phenotype and CD163 and CD206 for the M2 phenotype. Flow cytometry assay revealed that CD80 expression was upregulated in M0 phenotypes treated with oleamide (from 53.09% to 62.17%), whereas CD163 and CD206 were downregulated in M0 phenotypes treated with oleamide compared to the untreated control (from 58.50% to 42.10% and 63.85% to 56.24%, respectively). For the M1 phenotype, CD163 and CD206 expression levels were downregulated (from 10.72% to 5.36% and 74.14% to 54.20%, respectively), whereas CD80 was not differenced in M1 treated with oleamide compared to M1 untreated control. For the M2 phenotype, CD163 and CD206 expression levels were downregulated (from 85.32% to 59.67% and 86.04% to 71.03%, respectively), whereas CD80 was upregulated in M2 treated with oleamide compared to the control (58.21% to 64.60%) (**Figure 2**).

**Figure 3:**
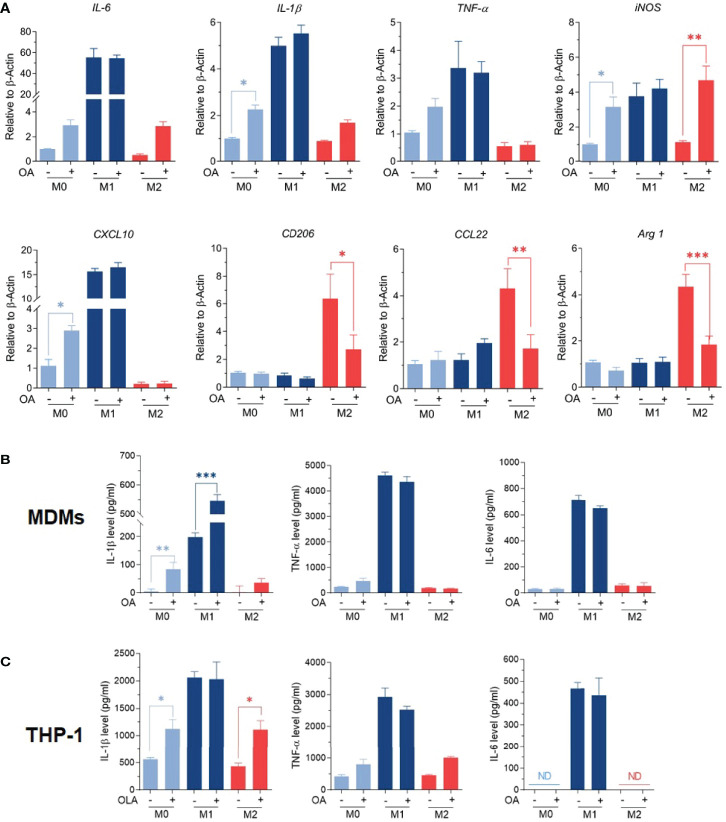
Oleamide mediated M1 macrophage polarization. **(A)** M1/M2 gene expression by RT-qPCR. **(B, C)** Cytokine levels by ELISA. MDMs (Day 6) or differentiated THP-1 were cultured in complete medium only (M0), LPS + INF-γ (M1), or IL-4 (M2) in the presence or absence of oleamide (15 µg/ml) for 24h in all experiments. One-way ANOVA was performed with multiple comparison corrections (Dunnett test). Data represent the mean ± SEM of three independent experiments. (*p < 0.05, **p < 0.01, ***p < 0.001).

M1/M2 phenotypes were further confirmed by studying the expression of M1-associated genes (*TNFα, IL-1β, IL-6, iNOS, CXCL10*), along with the expression of M2-associated genes (*Arg-1, CD206, and CCL22*) using RT-qPCR. Results showed that M0 macrophages treated with oleamide displayed increased expression of *TNF-α*, *IL-1β*, *IL-6*, and *iNOS* genes, while M2 macrophages treated with oleamide displayed defective expression of M2-associated genes (*Arg-1*, *CD206*, and *CCL22*) along with increased expression of the *IL-1β* gene. For M1 macrophages, treatment with oleamide displayed a minor change in both M1and M2-associated genes (**Figure 3A**). To support these results, pro-inflammatory cytokines IL-1β, IL-6, and TNF-α were determined by ELISA.

Interestingly, augment releasing of IL-1β was observed in all macrophage phenotypes after treatment with oleamide, whereas TNF-α and IL-6 increased in M0 macrophages but not significantly (**Figure 3B**). To confirm these results, cytokine production was further investigated using the THP-1 derived macrophage model. Surprisingly, increasing IL-1β production was also observed in M0 macrophages treated with oleamide, similar to the MDM model (**Figure 3C**). Therefore, this suggested that oleamide induced IL-1 β production in both MDMs and THP-1 cells. Taken together, these results indicated that oleamide mediated naïve macrophages (M0) toward the M1 phenotype and promoted IL-1β production while hindering the polarization of M2 phenotypes.

### Oleamide Induced IL-1β Production in LPS-Primed MDMs Involves Activation of the NLRP3- Inflammasome Pathway

**Figure 4:**
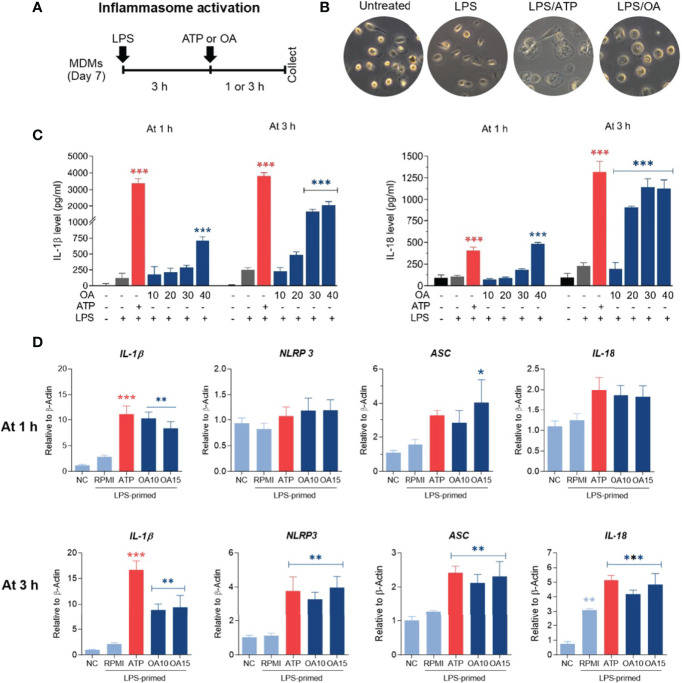
Oleamide mediated inflammasome activation and IL-1β and IL-18 production in LPS-primed MDMs. **(A)** Scheme of inflammasome activation. **(B)** Cell morphology by invested microscope. **(C)** IL-1β and IL-18 levels by ELISA. **(D)** Inflammasome-related gene expression by qRT-PCR. MDMs were primed with LPS (100 ng/ml) for 3 h followed by oleamide (OA) (15 µg/ml) or ATP (30 mM) for 1-3 h in all experiments. Data represents the mean ± SEM of three independent experiments (*p < 0.05, **p < 0.01, ***p < 0.001). NC, negative control; LPS, lipopolysaccharides; ATP, adenosine triphosphate; OA, oleamide; ASC, apoptosis-associated speck-like protein containing a C-terminal caspase recruitment domain; NLRP3, NOD-, LRR- and pyrin domain-containing protein 3.

An increase of IL-1β production was observed in both MDMs and THP-1 treated with oleamide, suggesting that oleamide functioned as a secondary signal to trigger NLRP3 inflammasome activation and lead to the production of IL-1β. To explore this hypothesis, naïve macrophages (M0) were primed with LPS (signal 1) followed by oleamide treatment for 1-3 h (**Figure 4A**). Then, cell morphology was observed using a bright-field microscope(**Figure 4B**). Cell-free supernatant was detected for IL-1β and IL-18 secretion and inflammasome-related genes (*IL-1β, IL-18, ASC, NLRP3*) were analyzed by RT-qPCR. As predicted, IL-1β and IL-18 were readily produced in LPS-primed MDMs exposed to oleamide in a dose- and time-dependent manner (**Figure 4C**). Furthermore, a high concentration of oleamide (30-40 µg/ml) induced IL-1β and IL-18 production equivalent to ATP, which is known as the NLRP3 inflammasome activation molecule. Consistent with results obtained using cell-free supernatant, oleamide induced upregulation of NLRP3, IL-1β, and IL-18 mRNA expression compared to the control cells (**Figure 4D**). Therefore, results suggested that oleamide mediated IL-1β and IL-18 production by triggering the NLRP3 inflammasome pathway.

### Oleamide Mediated IL-1β Production by Regulating NLRP3-Inflammasome in LPS-Primed MDMs

**Figure 5:**
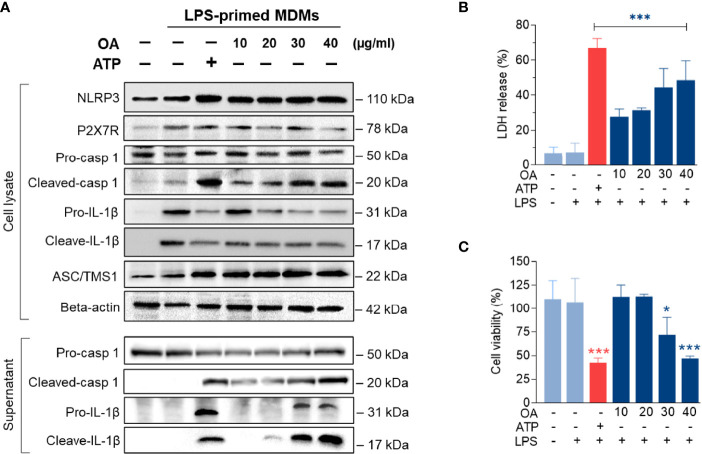
Oleamide promoted NLRP3 inflammasome activation in LPS-primed MDMs. **(A)** Western blot of whole cell lysate and supernatant from LPS-primed MDMs treated with oleamide (OA) (10 - 40 µg/ml) or ATP (30 mM) for 3 h** (B)** Release of LDH and **(C)** Cell viability (%) in LPS-primed MDMs treated with OA (10-40 µg/ml) or ATP (30 mM) for 3h Data represent the mean ± SEM of three independent experiments (*p < 0.05, ***p < 0.001). LPS, lipopolysaccharides; ATP, adenosine triphosphate; OA, oleamide; LDH, lactate dehydrogenase; ASC, apoptosis-associated speck-like protein containing a C-terminal caspase recruitment domain; NLRP3, NOD-, LRR- and pyrin domain-containing protein 3; P2X7R, purinergic P2X7 receptor.

To further investigate how oleamide impacted NLRP3 inflammasome activation and IL-1β production, LPS-primed MDMs were treated with oleamide for 3 h. Results showed that oleamide induced activation of NLRP3 and ASC speck formation similar to induction by ATP, which activated the NLRP3 inflammasome *via* the purinergic P2X7 receptor (**Figure 5A**). Both IL-1β and IL-18 are initially produced as biologically inactive pro-forms that require cleavage into mature cytokines. Typically, this processing is mediated by caspase-1, which is activated following the formation of an inflammasome (20). Our results showed that oleamide induced upregulation of cleaved caspase 1 and cleaved IL-1β in the supernatant, correlating to downregulation of these proteins in cell lysate (**Figure 5A**). The release of lactate dehydrogenase (LDH) induced by oleamide in LPS-primed MDMs significantly increased in a dose-dependent manner (**Figure 5B**), suggesting that oleamide increased caspase-1-mediated pyroptosis. Similar to LDH, an increase in cell death was observed in a dose-dependent manner (**Figure 5C**). These findings suggested that oleamide induced activation of the NLRP3 inflammasome pathway and mediated IL-1β production in LPS-primed MDMs.

## Discussion

Macrophage polarization is a process whereby macrophages adopt different functional phenotypes in response to specific microenvironmental stimuli and signals (2). This process is crucial for inflammation, tissue repair, and homeostasis maintenance (21). This study explored the immunoregulatory effects of oleamide focusing on macrophage polarization. Results indicated that oleamide mediated naïve macrophages (M0) toward the M1 phenotype and promoted NLRP-3 inflammasome-mediated IL-1β production in primary human monocyte-derived macrophages (MDMs) *in vitro*.

Macrophage polarization is conventionally subdivided into three groups as naïve macrophages (MØ; also called M0), which readily differentiate into the other two major phenotypes as classically activated macrophages (M1) and alternatively activated macrophages (M2) (1–3). Most research on macrophage polarization used simply *in vitro* techniques. Generally, macrophages derived from *in vitro* culture in the presence of specific cytokines stimulate M1 or M2 polarization. Here, M1 polarization was stimulated by LPS and IFN-γ, which are toll-like receptor (TLR) agonists. M2 polarization was stimulated by IL-4, designed to mimic what happens when macrophages are exposed to polarized CD4^+^ T cells, producing their distinctive cytokine combinations (3). M0 macrophages were cultured in complete media only without polarizing agents and considered naïve macrophages that had not been exposed to any pro- or anti-inflammatory stimuli or environment. This also avoids discrepancy from M-CSF or GM-CSF–dependent culture (22, 23). These three groups were cultured in the presence or absence of oleamide. The M0 and M2 macrophages displayed upregulation of CD80 coupled with downregulation of CD163 and CD206 on the cell surface after treatment with oleamide. In M1 macrophages, CD80 expression remained stable but displayed downregulation of CD163 and CD206 on the cell surface after treatment with oleamide (**Figure 2**). CD206 (also known as macrophage mannose receptor; MMR) is primarily expressed on the surface of macrophages and immature dendritic cells, where it acts as a pattern recognition receptor (PRR) (24, 25). Expression of CD206 is generally used as a marker of M2 macrophages (26–28). However, more than half of the M0, M1, and M2 phenotypes exhibited CD206 on the cell surface, indicating that using CD206 as an M2 marker in MDMs should be considered in terms of specificity.

Macrophage polarization is also characterized by the production of cytokines and the expression of M1/M2-associated genes. M1 macrophages are characterized by the production of proinflammatory cytokines like TNF-α, IL-1β, and IL-6. M2 macrophages are characterized by the production of IL-10 and TGF-β. Surprisingly, oleamide treatment did not alter the basal levels of IL-6 and TNF-α but specifically stimulated the production of IL-1β in both MDMs and differentiated THP-1 (**Figure 3C**). This indicated that oleamide might have some impact on IL-1β signaling and/or NLRP3 inflammasome.

IL-1β is a master regulator of inflammation by controlling a variety of innate immune cells (6, 8, 9, 29). In macrophages, inflammasome activation is required to process pro-IL-1β and pro-IL-18 into their mature forms and secrete active forms (IL-1β and IL-18), resulting in initiating inflammation. Currently, a two-step model is used for initiating inflammasome activation (6, 8, 9, 29). The first step or priming step (signal 1) typically involves an NF-κB-dependent upregulation of cellular NLRP3, pro-IL-1β, and pro-IL-18 protein synthesis. The second step (signal 2) is the activation of NLRP3 oligomerization. This step can be induced by numerous PAMP or DAMP such as extracellular ATP and K^+^efflux through the ATP-gated P2X7 channel, nigericin toxin as well as lysosomal destabilization agents (6, 8, 9). Upon activation, NLRP3 triggers self-oligomerization and recruitment of apoptosis-associated speck-like protein containing a caspase-recruitment domain (ASC) and pro-caspase-1, leading to the assembly of inflammasome complex (6, 8, 9). NLRP3 inflammasome activation results in active caspase-1, which cleaves the pro-IL-1β and pro-IL-18 into their mature forms, which then facilitates the robust immune responses and pyroptosis (6, 8, 9).

Dysregulation of NLRP3 inflammasome activation is linked with the development of many diseases, especially age-associated ailments such as neurologic disorders and metabolic diseases. Enhanced NLRP3 inflammasome-mediated IL-1β secretion in microglia is associated with the progression of Alzheimer’s disease by reducing the phagocytosis of Aβ from microglia (22, 30). The previous study by Yasuhisa Ano et al. reported that oleamide reduces amyloid-β (Aβ) accumulation *via* enhanced microglial phagocytosis, we hypothesized that oleamide could affect human macrophage NLRP3 inflammatory activation. Surprisingly, our research discovered that oleamide has a divisive effect on human macrophages. Results showed that oleamide mediated IL-1β and IL-18 secretion in LPS-primed MDMs nearly to ATP (**Figure 4C**). Moreover, oleamide induced activation of intracellular inflammasome-associated proteins including NLRP-3, ASC, cleaved casp-1and cleaved IL-1β, correlating with upregulation of cleaved IL-1β and cleaved casp-1 in the supernatant or secreted proteins (**Figure 5A**). However, no alteration of purinergic P2X7 receptor expression was observed after treatment with oleamide. This result indicated that oleamide-mediated NLRP-3 inflammasome activation was not involved in the activation of the P2X7 receptor. A previous study has been reported that P2Y type receptors, a family of G protein-coupled receptors, are potential targets of oleamide in primary murine microglia and human dendritic cells (30). P2Y type receptors involve the coupling of several intracellular pathways and second messengers but act more slowly than P2X receptors (22). However, the regulatory effect of oleamide *via* the P2Y receptor in the inflammasome activation has not been studied. Therefore, how oleamide regulates the NLRP3 inflammasome still requires further investigation. Furthermore, our findings were the first to show that oleamide can cause M1 macrophage polarization and inflammasome activation as well as IL-1β release in MDMs. However, the exact mechanism by which these released IL-1β regulate MDM polarizations is unknown.

Oleamide is an endogenous fatty acid amide that was first reported as a sleep-inducing substance and was later identified as having a wide range of receptors and neurotransmitter systems (15, 16, 23, 31). Nervous and immune systems are often cross-regulated and the potential immunoregulatory activity of oleamide was also studied. Oleamide suppressed the expression of iNOS and COX-2 and secretion of pro-inflammatory cytokines including TNF-α, IL-1β, and IL-6 in LPS-induced RAW264.7 murine macrophages (32). Surprisingly, this study found an opposite effect of oleamide that promoted the production of IL-1β and NLRP3 inflammasome activation in LPS-primed human MDMs. These opposite results were due to different experimental designs (pre-treatment vs. post-treatment), cell culture models (RAW264.7 cell vs. MDMs).

This study explored the immunoregulatory effects of oleamide on human macrophage polarization and NLRP3 inflammasome activation using primary human MDMs cultured in a medium containing human serum as a model. Our results indicated that oleamide promoted naïve macrophages (M0) toward the M1 phenotype and IL-1β production by regulating NLRP3 inflammasome activation in MDMs. This research shows that oleamide has a new effect on human MDMs and could be used as a therapeutic target for NLRP3-related inflammatory diseases, particularly neurodegenerative disorders.

## Conclusion

**Figure 6:**
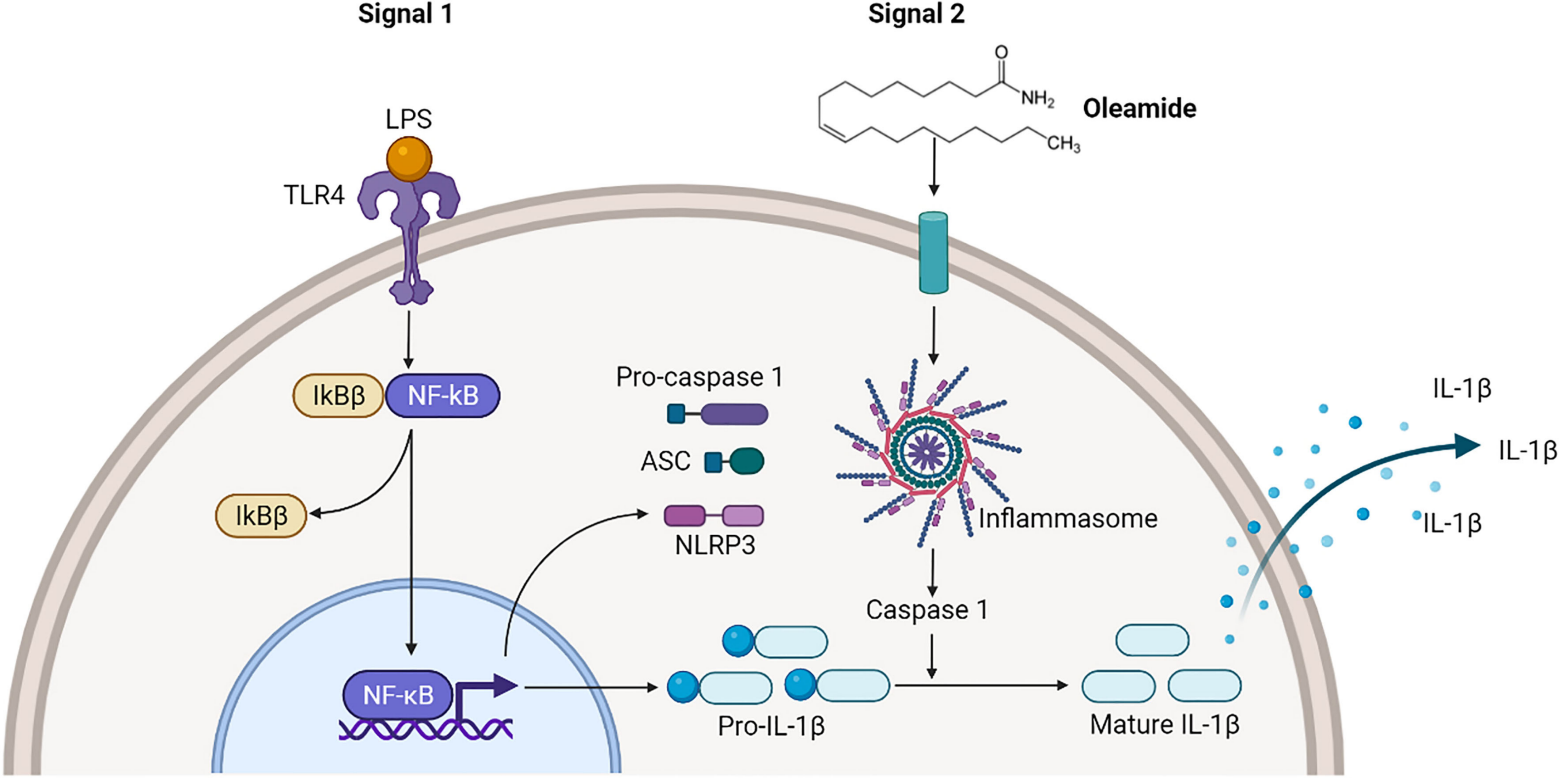
Oleamide activation of NLRP3 inflammasome of M1 phenotypes. The priming step (signal 1) by LPS involved an NF-κB-dependent upregulation of cellular NLRP3 and pro-IL-1β, while the second step (signal 2) was induced by oleamide activating inflammasome and cleaving IL-1β. The released IL-1β may help to induce the polarization of naive macrophages (M0) into M1 phenotypes by supporting oleamide.

Findings demonstrated that oleamide induced NLRP3 inflammasome activation and subsequently activated caspase 1, leading to the production of mature IL-1β, which secreted and promoted polarization of naive macrophages (M0) into M1 phenotypes (**Figure 6**). As a conclusion, oleamide could be a possible target for regulating the activation of the NLRP3 inflammasome and its implications for the treatment of inflammatory diseases.

## Data Availability Statement

The original contributions presented in the study are included in the article/**Supplementary Material**. Further inquiries can be directed to the corresponding author.

## Author Contributions

Conceptualization, PW and KU. Methodology, PW. Software, PW. Formal analysis, PW. Investigation, PW. Writing—original draft preparation, PW. Writing—review and editing, KU. Funding acquisition, KU and PP. Supervision, KU. Project administration, KU. All authors have read and agreed to the published version of the manuscript.

## Funding

This research was funded by the Royal Golden Jubilee Ph.D. Program (PhD/0002/2559), and the Thailand Science Research and Innovation, and Naresuan University (FF 2566).

## Conflict of Interest

The authors declare that the research was conducted in the absence of any commercial or financial relationships that could be construed as a potential conflict of interest.

## Publisher’s Note

All claims expressed in this article are solely those of the authors and do not necessarily represent those of their affiliated organizations, or those of the publisher, the editors and the reviewers. Any product that may be evaluated in this article, or claim that may be made by its manufacturer, is not guaranteed or endorsed by the publisher.
