# Supplementary material for: Oleamide-Mediated Polarization of M1 Macrophages and IL-1β Production by Regulating NLRP3-Inflammasome Activation in Primary Human Monocyte-Derived Macrophages
Source: Front Immunol. 2022 Apr 19;13:856296. doi: 10.3389/fimmu.2022.856296 (PMC9062104; doi:10.3389/fimmu.2022.856296)
Supplement: Supplementary file 1 [file DataSheet_1.pdf]

# *Supplementary Material*

**Supplementary Tables 1: Sequence of primers for qRT-PCR**

| Gene name | Forward primer (5'-3') | Reverse primer (5'-3') |
|---|---|---|
| *IL-1β* | AGCTACGAATCTCCGACCAC | CGTTATCCCATGTGTCGAAGAA |
| *TNF* | CCTCTCTCTAATCAGCCCTCTG | GAGGACCTGGGAGTAGATGAG |
| *IL6* | ACTCACCTCTTCAGAACGAATTG | CCATCTTTGGAAGGTTCAGGTTG |
| *CXCL10* | GTGGCATTCAAGGAGTACCTC | TGATGGCCTTCGATTCTGGATT |
| *CCL22* | CGAGGAAGAGGTTCGGTTCACC | CATCTTCACCCAGGGCACTCT |
| *CD206* | TCCGGGTGCTGTTCTCCTA | CCAGTCTGTTTTTGATGGCACT |
| *iNOS* | CAGGGTGTTGCCCAAACTG | GGCTGCGTTCTTCTTTGCT |
| *Arg1* | GTGGAAACTTGCATGGACAAC | AATCCTGGCACATCGGGAATC |
| *NLRP3* | ACAAACTCATGGTGGCTTCC | CGTGCATTATCTGAACCCCAC |
| *IL-18* | GAAGATGCCAGGGGTAATGA | TACCTGCCCCAAACTGAAAC |
| *PYCARD (ASC)* | TGACGGATGAGCAGTACCAG | AGGATGATTTGGTGGGATTG |
| *ACTB* | AGAAAATCTGGCACCACACC | CCATCTCTTGCTCGAAGTCC |

## 1.1 Supplementary Figures

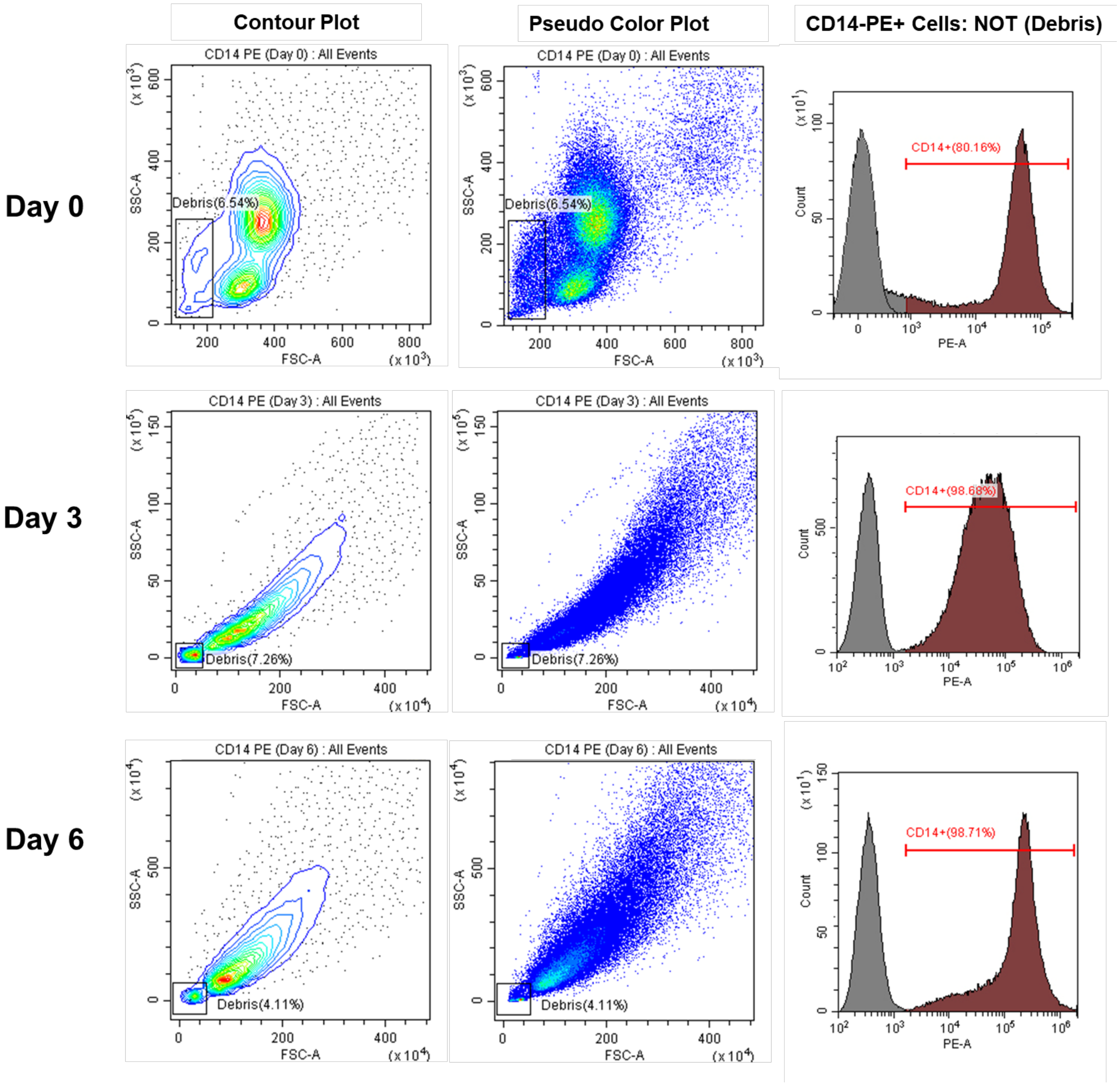


**Supplementary Figure 1. Purity of human MDMs at day 0, 3, and 6 by flow cytometry.** After isolation, human monocytes were cultured and differentiated in a complete medium for 6 days. Cell culture medium was replaced every 3 days. Percentage of CD14+ cells were analyzed at days 0, 3, and 6 as shown in the right diagram, which is the result of cell grading from all events except debris cells.

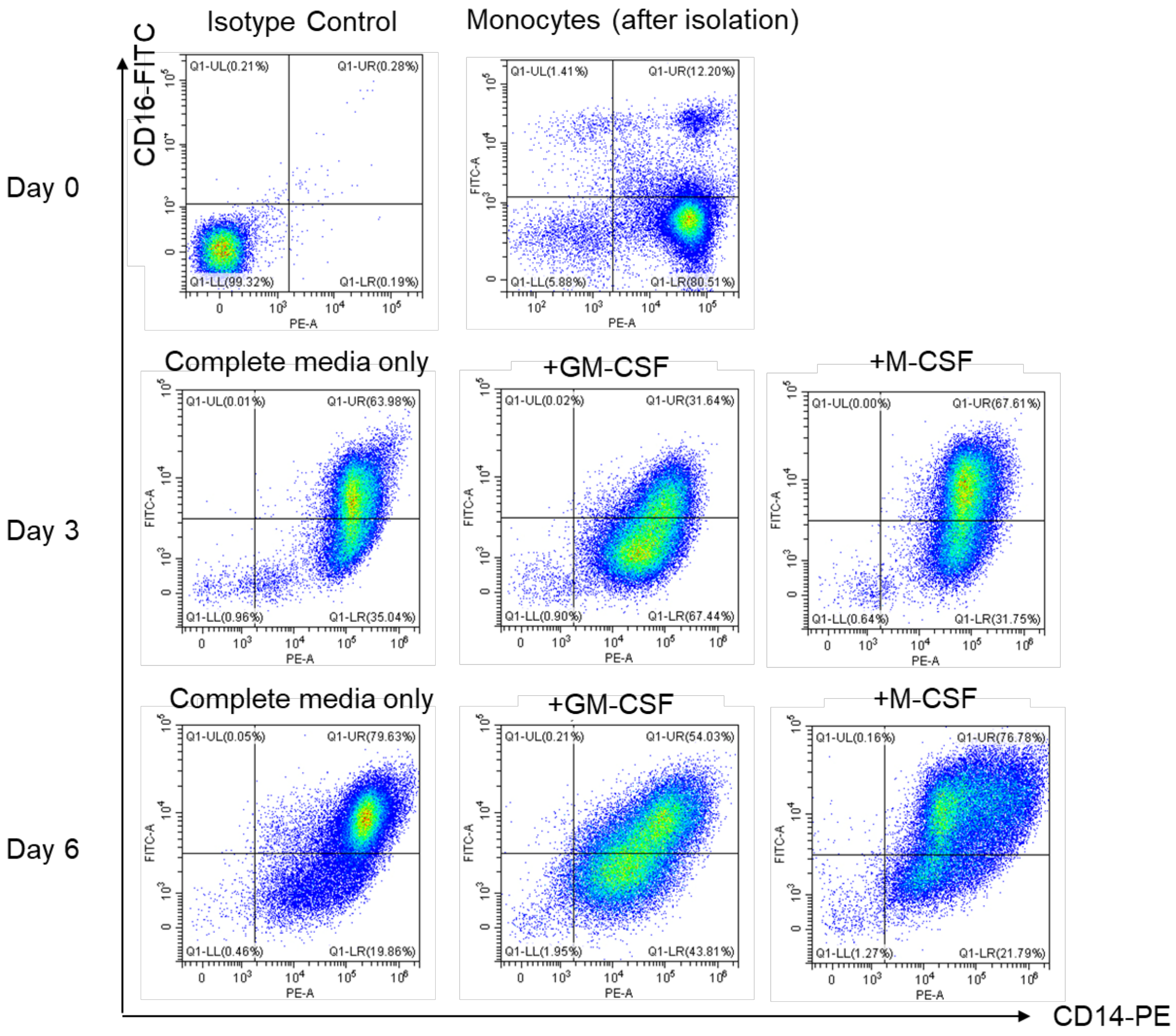


**Supplementary Figure 2. Analysis of CD14 and CD16 expression by flow cytometry.** Flow cytometry dot plot showing the gating of the CD14 PE and CD16 FITC in MDMs at days 0, 3, and 6. M0 cells were cultured in a complete medium only. M1-like cells were cultured in 50 µg/ml of GM-CSF while M2-like cells were cultured in 50 µg/ml of M-CSF. Cell culture mediums were replaced every 3 days.

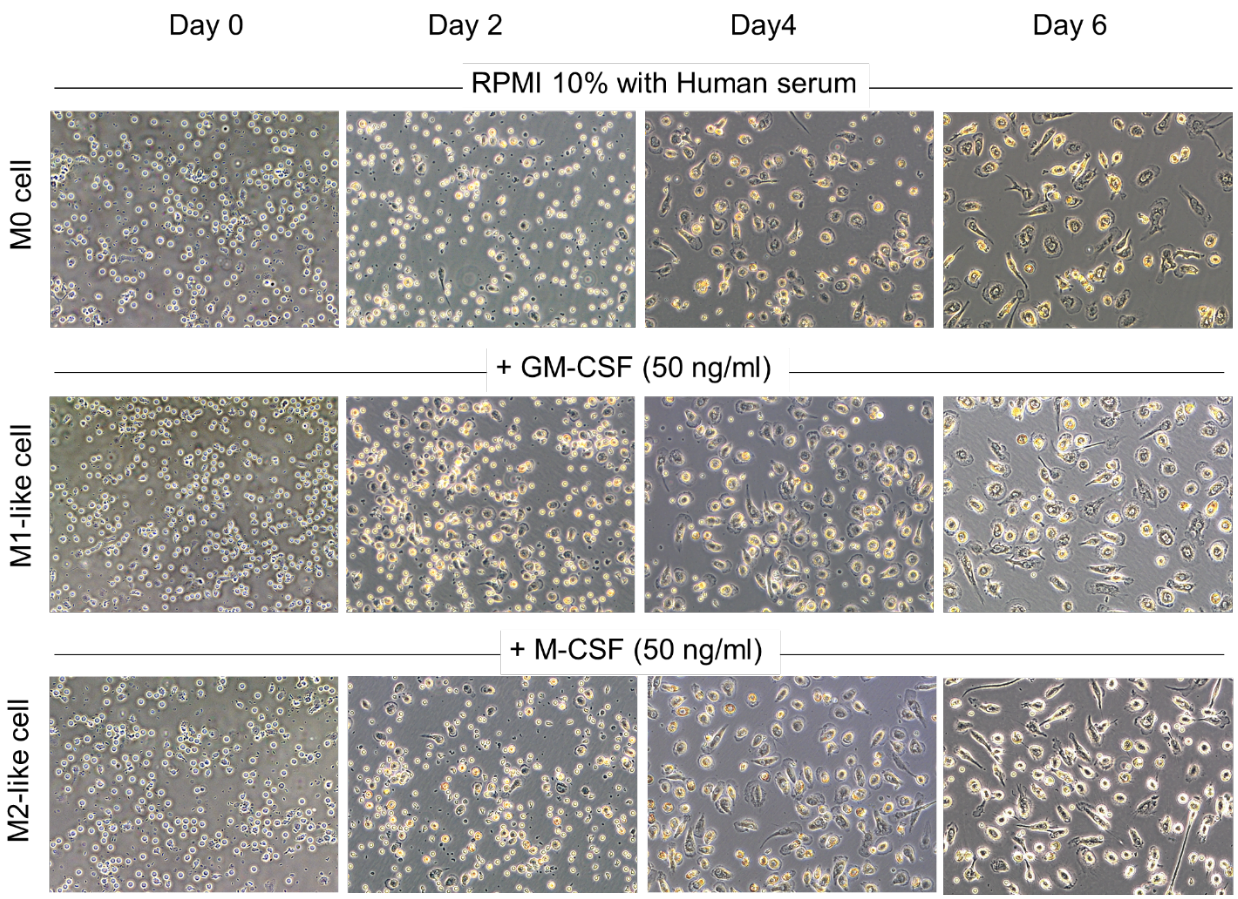


**Supplementary Figure 3. Culture and differentiation of MDMs at day 0-6.** M0 cells were cultured in a complete medium only. M1-like cells were cultured in GM-CSF. M2-like cells were cultured in M-CSF. (Magnification = 10X)

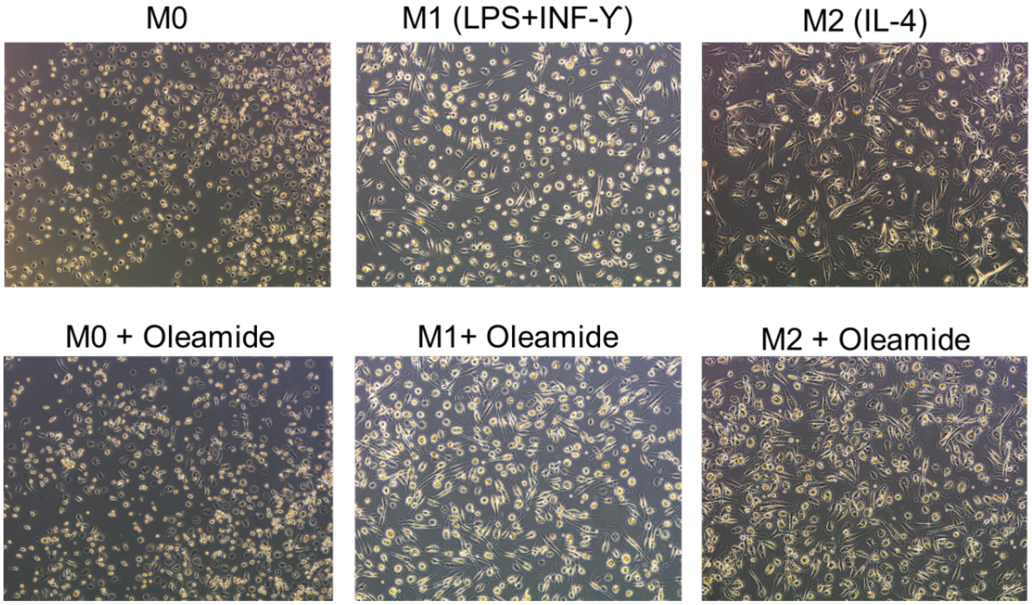


**Supplementary Figure 4. Morphology of polarized MDMs after treatment with oleamide.** At day 6, MDMs were cultured in complete medium only (M0), LPS + INF-γ (M1), or IL-4 (M2) in the presence or absence of oleamide (15 μg/ml) for 24h. (Magnification = 10X)

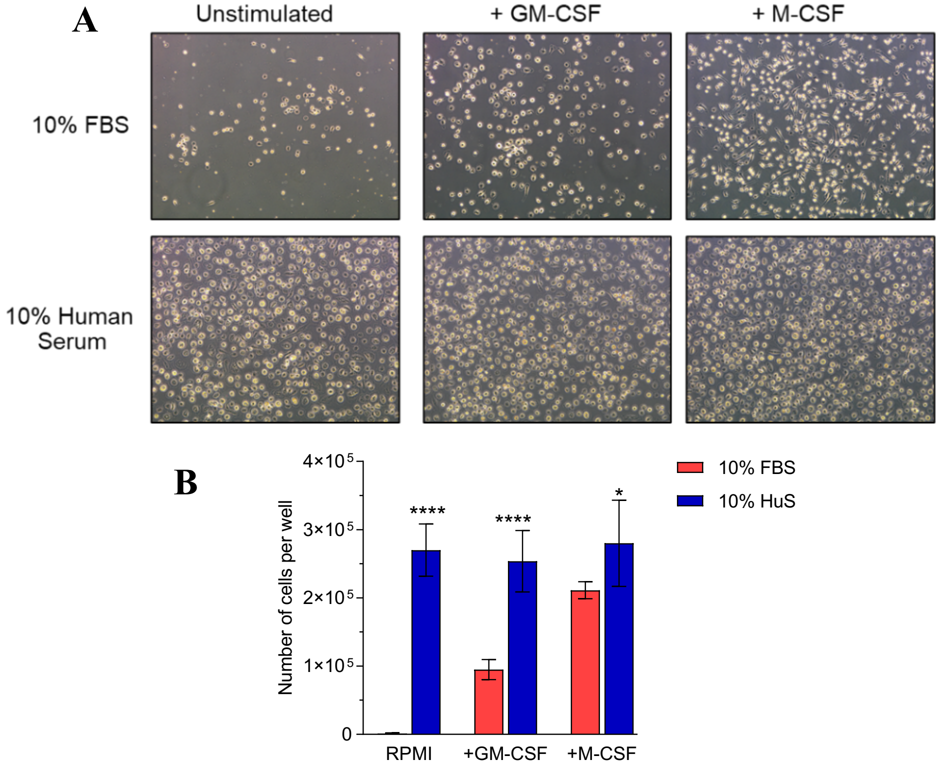


**Supplementary Figure 5. Comparison of MDMs cultured in medium containing 10%FBS VS 10%human serum.** (A) Morphology of MDM and (B) Number of adherence cells per well at day 6. MDMs were cultured in complete medium (10% FBS or 10% Human serum, HuS) in the presence or absence of GM-CSF or M-CSF for 6 days.

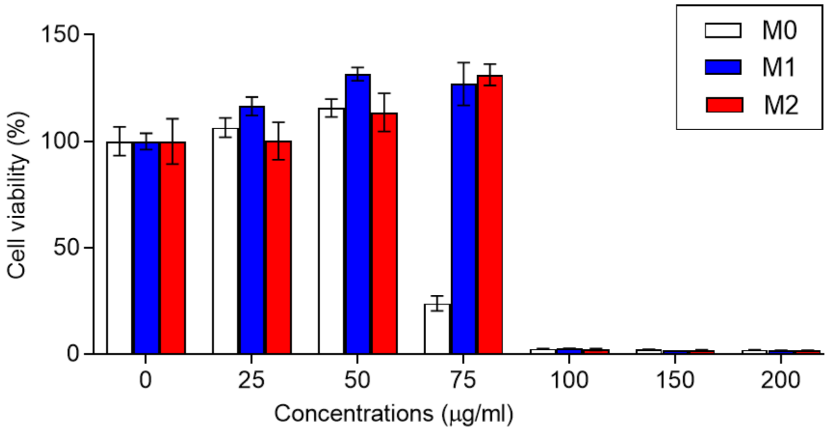


**Supplementary Figure 6. Cell viability (%) of MDMs after treatment with oleamide.** Polarized MDMs (M0, M1, and M2 macrophages) were incubated with a serial concentration of oleamide for 48 h.
